# Differences in the Disaster-Preparedness Behaviors of the General Public and Professionals: Evidence from Sichuan Province, China

**DOI:** 10.3390/ijerph17145254

**Published:** 2020-07-21

**Authors:** Zhuolin Yong, Linmei Zhuang, Yi Liu, Xin Deng, Dingde Xu

**Affiliations:** 1 College of Management, Sichuan Agricultural University, Chengdu 611130, China; zhuolinyong@stu.sicau.edu.cn (Z.Y.); zhuanglinmei@stu.sicau.edu.cn (L.Z.); lyx1@stu.sicau.edu.cn (Y.L.); 2 College of Economics, Sichuan Agricultural University, Chengdu 611130, China; dengxin@sicau.edu.cn; 3 Sichuan Center for Rural Development Research, College of Management, Sichuan Agricultural University, Chengdu 611130, China

**Keywords:** disaster preparedness, professionals, general public, earthquake, Sichuan Province, China

## Abstract

Sichuan Province in China is one of the world’s most earthquake-stricken areas. Wenchuan and Lushan Counties in Sichuan and other earthquake-stricken areas contain rural settlements subject to geological disasters and poverty. However, there is little research on the characteristics of disaster-preparedness behavior and whether these differ between professionals and the general public in rural settlements with high earthquake risk and poverty. Using survey data from 327 farmers in rural settlements affected by major earthquakes in Wenchuan and Lushan Counties, independent-sample t-tests and chi-squared tests were used to test for differences in the disaster-preparedness behaviors of professionals and the general public. The results show that (1) there were significant differences in emergency-disaster preparedness, knowledge and skills preparedness and overall disaster-prevention preparedness, and (2) there was no significant difference in physical disaster-prevention preparation. Based on these results, the study suggests policy directions for regional poverty alleviation, disaster prevention and reduction and disaster management.

## 1. Introduction

The primary task of the rural-revitalization strategy of the 19th National Congress of the People’s Republic of China (CPC) is to alleviate poverty and achieve prosperity for the poor by 2020 [1,2,3,4,5,6,7,8]. The 2019 central government document No. 1, “Prioritizing the development of agriculture and rural areas, and assisting agriculture, rural areas and farmers”, clearly states that in order to promote rural development in a down-to-earth manner, it is necessary to speed up the completion of rural human settlements and public services [9,10,11]. Since the 18th National Congress of the CPC, China has made remarkable achievements in poverty alleviation [12]. The number of poor people in rural areas has decreased from 98.99 million to 17.13 million from 2012 to 2018—an average of nearly 14 million per year. However, by the end of 2018, there were still more than 17 million people living in poverty in rural areas—mainly in 14 contiguous impoverished mountainous areas [13,14]. Due to geology, topography and the level of local economic development, some settlements in these areas have a relationship between geological disasters and poverty [15,16]. Sichuan is a world-famous major earthquake area and is China’s most serious earthquake disaster province [3,4]. From 2000 to 2017, 179 earthquakes of magnitude five or above occurred in China, resulting in a total of 488,437 casualties, of which 94.17% were in Sichuan Province. The direct economic losses from earthquake disasters in China amounted to 1133.4 billion Yuan, of which 938.7 billion Yuan or 82.82%, was in Sichuan Province [17]. The Wenchuan earthquake and Lushan earthquake are two major earthquakes with a magnitude of more than seven that caused about 450,000 casualties and direct economic losses of more than 900 billion Yuan [18,19]. Due to geological disasters and extreme weather conditions, poverty-prone populations in some Western mountain areas have returned to poverty at an average rate of 15–25%—or as high as 30–50% in some places [20,21]. Unlike the poverty effects caused by an extreme climate, geological disasters have the characteristics of occurring suddenly and being highly destructive [22,23]. The life savings of farmers can be wiped out instantly by such disasters, which have become a major way that farmers enter poverty or return to it after being previously lifted out of it [24,25]. Therefore, in the context of the “affluent life” proposed in the “rural revitalization strategy”, and to achieve the goal of have a prosperous poor population by 2020, it is necessary to carry out research on the large group of farmers that live in regions prone to geological disasters and poverty.

When dealing with earthquake disasters in high-risk earthquake areas, adequate disaster-avoidance preparation can reduce the threats to life and property loss and, thus, reduce the likelihood that farmers will return to poverty [26,27,28,29]. Many empirical studies have shown that residents’ disaster preparedness is an effective means to deal with disaster shocks [30,31,32,33,34,35]. Disaster preparedness refers to the measures taken by residents to avoid disasters [36,37,38]. Different studies have different entry points for the measurement of residents’ preparedness to avoid disasters [39,40,41,42]. Existing research has focused on protecting residents’ life safety by measuring their disaster preparedness (e.g., [32,33,34]). Some scholars think that residents’ preparedness for avoiding disasters is a one-dimensional concept, which can be directly measured by asking residents whether they have made adequate preparedness for avoiding disasters (e.g., [43]). Some scholars think that residents’ preparedness for disaster avoidance is a multi-dimensional concept, which can be measured from multiple dimensions. For example, Onuma et al. [44] divided residents’ preparedness for avoiding disasters into three dimensions of basic preparedness, energy/heat preparedness and assessment of specific design indicators for each dimension. At the same time, many studies believe that the residents’ preparedness for avoiding disasters is simply material preparation, which is measured from the perspective of residents’ material preparation. Among them, the most common way to measure disaster preparedness is to ask residents whether they have prepared emergency disaster kits (flashlights, radios, clean water) (e.g., [35,36,45,46]), buy insurance (e.g., [24,25]), if they had an escape plan (e.g., [39,40,41,42,47,48]) and reinforce houses (e.g., [38,49]). In addition, some scholars have noted that in addition to material preparation, residents’ disaster preparedness, conscious disaster risk management behavior is also an effective means to deal with disaster impact (e.g., [21]). In general, the existing research provides useful reference for the measurement of disaster preparedness in this study. however, few research systems comprehensively consider three dimensions of emergency disaster preparedness, knowledge and skills preparedness and physical disaster prevention and set indicators to conduct systematic measurement of residents’ behavior of disaster preparedness. In the face of an earthquake disaster, a family’s disaster preparedness (such as preparing an emergency box) can increase their safety to some extent, but assistance for the reconstruction of their family life is limited. Determining the best way to construct a complete, efficient and resilient disaster prevention and control system that makes up for the shortcomings of current systems is of great significance to rural revitalization. It is a topic of high relevance to residents and governments of disaster areas and to society in general. Therefore, compared with the existing research, one of the marginal contributions of this research is to integrate the dimensions of residents’ preparedness for earthquake disaster and measure it with specific indicators.

To date, a large number of studies have focused on earthquake disasters and to explore their disaster-preparedness behaviors and their influences (e.g., [40,41,50]). According to the general logic, if a region is well prepared for disaster, there should be no significant difference in preparedness between general public and professionals [42]. However, judging from the reality of disaster threat zones around the world, professionals tend to be better prepared than the general public. Identify the significant difference in disaster preparedness between professionals and the general public has two benefits, on one hand, it is helpful to identify the shortcomings of disaster preparedness of general public and formulate relevant policies to improve their disaster prevention and reduction capacity; on the other hand, we can find out the deficiencies in disaster preparedness of professionals and formulate corresponding policies to improve regional disaster prevention and reduction capacity. However, few studies have further analyzed the characteristics and differences of disaster preparedness between professionals and the general public. Professionals are generally full-time village cadres who have been systematically trained by professional institutions such as the state land administration (In China, the Land and Resources Administration of The People’s Republic of China will organize professionals to participate in the training program of mass monitoring and mass prevention systems, systematically introduce experience in disaster prevention and reduction, and conduct some on-site disaster risk management exercises, so that professionals can better guide the general public in disaster risk management), and have the ability to protect their own lives and property, as well as the professional ability to observe the signs of earthquakes and warn the general public in a timely manner. The general public are local farmers in high-risk areas who do not have sufficient disaster awareness and preparedness. Compared with other studies, the second marginal contribution of this study is that the research objects are divided into professionals and general public, and the characteristics and differences of disaster preparedness of the two groups are systematically analyzed. Based on this, the objective of this study is to analyze the characteristics and differences of disaster preparedness between professionals and general public through the survey data of farmers in China’s earthquake-stricken areas, so as to provide references for the formulation of regional disaster prevention and reduction policies.

Under the above background, this study focuses on the characteristics and differences of disaster preparedness between general public and professionals, in order to provide a reference for the formulation of policies related to poverty alleviation and disaster risk management of rural households in areas where earthquake disaster and poverty are intertwined. There are two key problems to be solved in this study:

(1) One is to analyze the characteristics of disaster preparedness of general public and professionals;

(2) The second is to compare whether there is a significant difference between general public and professionals in disaster preparedness. If there is, further analysis will be made on the difference between the two groups in disaster preparedness.

### Theoretical Basis and Variable Measure

Based on the above analysis of introduction, referring to the disaster risk management literature, this study measures disaster-preparedness behavior in three dimensions: (1) emergency-disaster preparedness, (2) knowledge and skills preparedness and (3) physical disaster-prevention preparedness (Table 1). Among them, the selection of emergency-disaster preparedness dimension measurement indicators is based on references [35,36,45,46], the selection of knowledge and skills preparedness dimension measurement indicators is based on references [24,25,38,49] and the selection of knowledge and skills preparedness dimension measurement indicators is based on references [21,24].

Generally speaking, if a region is well prepared in disaster risk reduction and if disaster-related knowledge is well publicized, there should be no significant difference in disaster preparedness between professionals and the general public. Therefore, the following research assumptions were made in this study:

**H1:**  *There is no significant difference between the general public and professionals in their three dimensions of general disaster-preparedness behaviors (emergency-disaster preparedness, knowledge and skills preparedness, and physical disaster-prevention preparedness)*.

## 2. Materials and Methodology

### 2.1. Introduction to the Research Area

Sichuan province is one of the most earthquake-prone areas in China, the most famous of which are the Wenchuan earthquake in 2008 and the Lushan earthquake in 2012 [16]. According to statistics, the Wenchuan earthquake in 2008, with a magnitude of 8.0, destroyed more than 100,000 square kilometers and involved 237 districts and counties, causing nearly 460,000 casualties and direct economic losses of up to 845.215 billion Yuan [19]. With a magnitude of 7.0, the Lushan earthquake in 2012 mainly affected Lushan county, Baoxing County, Tianquan County and other counties, and affected a total of 383,000 people, with direct economic losses of up to 50 billion Yuan [16,19].

### 2.2. Data Collection and Sampling Methods

**Figure 1:**
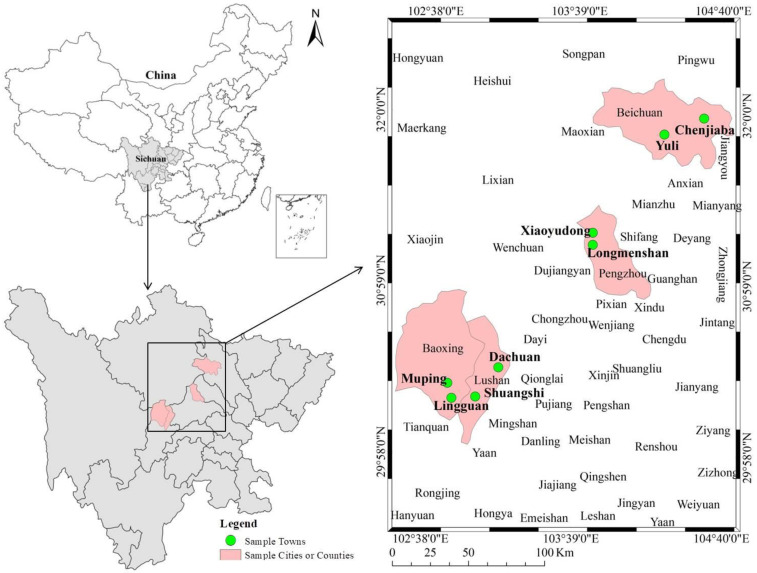
Distribution of survey counties and towns.

The data for this study were mainly obtained from a questionnaire survey conducted by the research team at Sichuan Agricultural University in July 2019 in the areas hardest hit by the 2008 Wenchuan earthquake and the 2012 Lushan earthquake. The research method was one-on-one face-to-face interviews, which mainly focused on residents’ disaster risk perceptions and behavior choice of disaster preparedness. Each interview lasted about 1.5 h. In order to ensure the typicality and representativeness of the samples, a stratified random sampling method was used to collect sample survey data [24,51,52], as follows:

First, according to the situation of threatened population and social and economic development in the worst-hit areas in Wenchuan earthquake and Lushan earthquake, the surveyed sample areas and counties were determined. Pengzhou and Beichuan Qiang Autonomous County were selected as the sample counties (cities) that representing the ten Wenchuan earthquake—hit areas and counties, while Lushan and Baoxing County were selected representing the six Lushan earthquake—hit areas and counties. Second, according to the differences in the severity of the disaster, economic development levels of the districts and the distances from the centers of the districts, and two sample towns were randomly selected for each sample districts, resulting in a total of eight towns. Third, the study identified 16 sample villages (8 sample towns randomly selected 2 sample villages each) with similar indices and methods. Finally, according to the random number table set by the team and the list of farmers obtained from the village cadres, 20–23 households were randomly selected from each sample village as the sample farmers for the survey. After strict training, 13 researchers, led by village cadres, conducted one-on-one face-to-face surveys at the farmers’ homes. In the end, a total of 327 valid questionnaires were obtained from 16 villages and eight towns in four districts and counties (Figure 1). Among them, thirty-four are professionals and the rest are members of the general public.

### 2.3. Methods of Data Analysis

This study attempts to explore the differences of disaster-preparedness behavior between general public and professionals in terms of emergency-disaster preparedness, knowledge and skills preparedness and physical disaster-prevention preparedness. The statistical analysis methods include descriptive statistical analysis, mean difference analysis of continuous variables in different categories (independent sample t-tests) and difference analysis of binary variables (chi-squared tests).

## 3. Results

### 3.1. Differences in Emergency-Disaster Preparedness

As can be seen from Table 2, in terms of the types of emergency supplies prepared, there were significant differences between general public and professionals in six specific items: water, food, emergency lights, first aid kits and manuals, important documents and cash and clothes. In the wake of a disaster, there were new and significant differences in ownership of radios, fire extinguishers, special items (such as medicine) and other items. In addition, the mean value, standard deviation and significant difference of these variables were significantly higher after a disaster than before one, indicating that the occurrence of a disaster prompts farmers to prepare disaster-avoidance items and enhance their awareness.

### 3.2. Differences in Knowledge and Skills Preparedness

As can be seen from Table 3, while there was no significant difference between the two groups in their access to knowledge, there was a significant difference between the two specific ways in which they obtained paper-based seismic popular science data and accessed the websites of seismological departments.

### 3.3. Differences in Physical Disaster-Prevention Preparedness

As can be seen from Table 4, there was no significant difference between general public and professionals in physical disaster preparedness, among which the two indicators of *building reinforcement* and placing of important items were not significantly different. Some 59% of professionals reinforced their houses (3% higher than the general population), possibly because of the high cost of effective housing reinforcement and the low economic capacity of farmers in high-risk earthquake areas. This limits the opportunity for farmers to reinforce their houses. Some 59% of professionals kept important items in a safe place (4% less than the general population), probably due to the general high awareness of property protection among farmers.

### 3.4. Overall, Disaster Preparedness Differences

It can be seen from Table 4 that there was a significant difference in the disaster-preparedness behaviors of farmers (general public and professionals) in poverty-stricken, earthquake-prone regions, which is not consistent with the hypothesis.

As can be seen from Table 4, there was a significant difference between general public and professionals in *emergency-disaster preparedness*, which is reflected in the *preparation of emergency goods* and *disaster prevention and mitigation* measures, but there was no significant difference in whether or not they purchased natural disaster insurance. Specifically, 56% of professionals had emergency supplies, which is 35% higher than the general public; 94% of professionals had taken preventive measures, which is 21% higher than the general public. However, only 27% of the general public had taken out natural disaster insurance, which is almost the same rate as for professionals. This may be due to a low awareness of disaster risk among local farmers and the low amount of insurance coverage offered, which results in low enthusiasm to take out insurance.

It can be seen from Table 4 that there were significant differences between general public and professionals in knowledge and skills preparation, which are reflected in independent learning, training and escape drills, while there were no significant differences in knowledge acquisition channels. Specifically, 85% of professionals participated in disaster prevention skills training (44% more than the general public); and 88% of professionals participated in escape drills organized by the village (36% more than the general public). The proportion of professionals with access to knowledge was 97%, while that of the general public was 88%. The difference was not significant, possibly because the survey sites were all in the main earthquake area or area of influence, and the local village committee provided sufficient access to earthquake disaster knowledge to ensure that most people could access relevant knowledge.

## 4. Conclusions

Through the previous analysis, we get the following main conclusions:

(1) There were significant difference between the general public and professionals in emergency-disaster preparedness, knowledge and skills preparedness and overall disaster-prevention preparedness;

(2) There was no significant difference between the general public and professionals in physical disaster-prevention preparedness.

## 5. Policy Implications

The disaster and poverty situation in Sichuan Province is quite severe and many large earthquakes have occurred, which has aroused wide public concern. This is also one of the most important challenges in poverty alleviation in the southwestern mountainous areas. Therefore, earthquake-related disaster prevention and mitigation work is particularly important, even though most earthquake-prone areas are equipped with relevant professionals. However, there is a significant difference between professionals and the general public in disaster-preparedness behavior. Based on the above conclusions, the following countermeasures and suggestions are proposed for disaster prevention and reduction by farmers in areas where earthquakes and poverty are interwoven.

(1) Professionals should strengthen their awareness of insurance participation and disaster crises. Professionals have rich knowledge reserves for the cognition and perception of earthquake disasters and have relevant scientific literacy that helps in the understanding of disaster-avoidance articles, escape behavior and reducing loss of life and property after disasters. However, they have some shortcomings in their awareness and purchasing of natural disaster insurance, reinforcement of houses and placement of important items. The government could start by raising the awareness of insurance professionals themselves and general awareness of disaster crises. This could be strengthened by advising insurance companies to increase their types and amounts of natural disaster insurance offered, increase the publicity of natural disaster insurance, and increase subsidies for insurance and house reinforcement.

(2) The general public should increase their initiatives in disaster prevention and preparedness. There are significant differences between the emergency-disaster preparedness and the knowledge and skills preparedness of the general public in the face of disasters, especially in the following five aspects: emergency supplies preparedness, preparedness for preventive measures, self-study and participation in training and escape drills. The difference between the two groups in these five aspects is, in the final analysis, the difference between a subjective initiative in disaster preparedness and a sense of disaster avoidance. The government may consider promoting the initiative of the general public to avoid disasters. This can be done by promoting education on disaster prevention, establishing grassroots disaster prevention groups and establishing village disaster-prevention preparedness and supervision committees.

(3) The government’s disaster prevention policy-making should expand its audience appropriately. The government formulates disaster prevention policies and measures and systematically trains full-time village cadres as professionals for the ultimate purpose of ensuring that there is no significant difference between professionals and the general public in all aspects of disaster prevention and reduction. However,—according to the research results—the overall difference between the two groups is still significant, indicating that the range of people reached by existing policies and measures is small. In future policy formulation, the audience should be expanded appropriately to make up for the shortcomings of the general public in disaster prevention and mitigation, so as to eliminate the significant difference between the two groups.

(4) The system of settlement disaster prevention and reduction should be improved. The government can jointly build a disaster prevention and mitigation system for settlements based on the three aspects of emergency-disaster preparedness, knowledge and skills preparedness and physical disaster prevention. It should give full play to the leading and exemplary role of professionals in emergency-disaster preparedness and knowledge and skills preparedness, actively organize knowledge-based training on natural disasters such as earthquakes, organize regular earthquake escape drills, regularly inspect the soundness of houses prepared by farmers for disaster mitigation, and give special disaster-prevention guidance to poor farmers. We will appropriately increase subsidies for settlement in mountainous areas, improve farmers’ production conditions and transportation environment; strengthen farmers’ own capacity to consolidate the achievements of poverty alleviation.

## Figures and Tables

**Table 1:** Measures of preparation behavior for earthquake disaster avoidance.

Category	Variable	Measure Indicators (0 = No, 1 = Yes)
Emergency disaster preparedness	Emergency supplies	Does your home usually prepare disaster emergency supplies?
	Natural disaster insurance	Does your family have enough natural disaster insurance?
	Preventive measures	Is your family taking preventive measures?
Knowledge and skills preparedness	Autonomous learning	Do family members regularly learn disaster risk reduction techniques?
	Knowledge acquisition channel	Is there access to disaster information?
	Training	Has anyone in your family been trained in disaster knowledge?
	Escape drills	Has anyone in your family ever participated in the disaster escape drill organized by the village?
Physical disaster-prevention preparedness	Building reinforcement	Is the home reinforced?
	Important item placement	Do you keep important items in a safe place?

**Table 2:** Differences in disaster-preparedness items between the general public and professionals before and after disasters.

Variable	Before Disaster		After Disaster		Significant Difference before Disaster	Significant Difference after Disaster
	Mean	S.D.	Mean	S.D.		
Water	0.08	0.27	0.23	0.42	−0.144 ***	−0.331 ***
Food	0.09	0.28	0.21	0.41	−0.134 ***	−0.286 ***
Emergency light	0.04	0.20	0.17	0.38	−0.116 ***	−0.301 ***
Radio	0.00	0.00	0.02	0.13	0.000	−0.045 *
First aid kit and manual	0.01	0.08	0.06	0.23	−0.059 ***	−0.198 ***
Fire extinguisher	0.01	0.08	0.06	0.23	0.007	−0.136 ***
Special items (e.g., medicine)	0.02	0.13	0.11	0.32	0.020	−0.136 **
Important documents and cash	0.01	0.11	0.08	0.27	−0.052 ***	−0.210 ***
Clothes	0.04	0.19	0.16	0.36	−0.090 ***	−0.351 ***

Note: *, **, *** are significant at the levels of 0.1, 0.05 and 0.01, respectively.

**Table 3:** Differences in knowledge and skills preparedness between the general public and professionals.

Access to Knowledge	People in General		Mean of the General Public	Mean of Professionals	Significance
	Mean	S.D.			
News media	0.80	0.40	0.80	0.79	0.005
Paper seismic science materials	0.41	0.49	0.39	0.62	−0.232 ***
Earthquake department website	0.06	0.25	0.05	0.21	−0.158 ***
School education	0.15	0.35	0.14	0.18	−0.033
WeChat/Weibo	0.29	0.45	0.29	0.29	−0.004
Promotion of government	0.12	0.32	0.11	0.18	−0.064
No channel	0.09	0.29	0.10	0.06	0.037

Note: *** are significant at the levels of 0.01.

**Table 4:** Differences in disaster preparedness between the general public and professionals.

Category	Variable	General Public		Professionals		Significance
		Mean	S.D.	Mean	S.D.	
Emergency-disaster preparedness	Emergency suppliers	0.21	0.41	0.56	0.50	0.000 ***
	Natural disaster insurance	0.27	0.45	0.29	0.46	0.794
	Preventive measures	0.73	0.44	0.94	0.24	0.007 ***
Knowledge and skills preparedness	Autonomous learning	0.58	0.49	0.91	0.29	0.000 ***
	Knowledge acquisition channel	0.88	0.32	0.97	0.17	0.112
	Training	0.41	0.49	0.85	0.36	0.000 ***
	Escape drill	0.52	0.50	0.88	0.33	0.000 ***
Physical disaster-prevention preparedness	Building reinforcement	0.56	0.50	0.59	0.50	0.780
	Important items placement	0.63	0.48	0.59	0.50	0.622
Emergency-disaster preparedness		0.41	0.28	0.60	0.29	0.0002 ***
Knowledge and skills preparedness		0.60	0.31	0.90	0.22	0.0000 ***
Physical disaster-prevention preparedness		0.60	0.35	0.59	0.31	0.8845
Overall disaster-prevention preparedness		0.53	0.23	0.70	0.20	0.0001 ***

Note: *** is significant at the levels of 0.01.
